# The prevalence of cardiovascular autonomic neuropathy and its influence on post induction hemodynamic variables in patients with and without diabetes; A prospective cohort study

**DOI:** 10.1371/journal.pone.0207384

**Published:** 2018-11-26

**Authors:** Jorinde A. W. Polderman, Nicolaas H. Sperna Weiland, Michel H. Klaver, Judy Biginski, Marijn Horninge, Markus W. Hollmann, J. Hans DeVries, Rogier V. Immink, Benedikt Preckel, Jeroen Hermanides

**Affiliations:** 1 Department of Anesthesiology, Amsterdam UMC, University of Amsterdam, Amsterdam, the Netherlands; 2 Department of Endocrinology, Amsterdam UMC, University of Amsterdam, Amsterdam, the Netherlands; Public Library of Science, UNITED KINGDOM

## Abstract

**Background:**

Cardiovascular autonomic neuropathy (CAN) is a known complication of diabetes, but is also diagnosed in patients without diabetes. CAN may be related to perioperative hemodynamic instability. Our objective was to investigate if patients with diabetes would have a higher prevalence of CAN compared to patients without diabetes undergoing surgery. We further studied its relation to changes in post-induction hemodynamic variables.

**Methods:**

We prospectively included 82 adult patients, 55 with DM, 27 without DM, scheduled for major abdominal or cardiac surgery. Patients performed four autonomic function tests on the day before surgery. Primary outcomes were the prevalence of CAN and the relation between CAN and severe post-induction hypotension, defined as mean arterial pressure (MAP) < 50 mmHg or ≥ 50% decrease from baseline. Secondary outcomes were the relation between CAN, intraoperative hypotension, MAP < 65 mmHg for more than 13 minutes, and the use of vasopressor therapy.

**Results:**

The prevalence of CAN in patients with or without DM was 71% versus 63%, (p = 0.437). CAN was not associated with severe post induction hypotension (CAN+ vs. CAN–: 21% vs. 19.2%, p = 0.819) nor with intraoperative hypotension (16% vs. 15%, p = 0.937). Patients with definite CAN received more norepinephrine in the perioperative period compared to patients with mild CAN or no CAN (0.07 mcg kg^-1^ min^-1^ (0.05–0.08) vs. 0.03 (0.01–0.07) vs. 0.02 (0.01–0.06) respectively, p = 0.001).

**Conclusions:**

The majority of patients studied had mild to moderate CAN, regardless of the presence of DM. Assessing CAN before surgery did not identify patients at risk for post induction and intraoperative hypotension in our cohort.

**Trial registration:**

Dutch Trial Registry (www.trialregister.nl) NTR4976.

## Introduction

Cardiovascular autonomic neuropathy (CAN) is characterized by an imbalance of the parasympathetic and sympathetic tone resulting in loss of heart rate variability, resting tachycardia, orthostatic hypotension and sudden death.[1–3] One-third of the postoperative complications is due to a cardiac event.[4] Perioperative hypotension has been related to myocardial injury after non-cardiac surgery.[5] Only few studies have assessed the influence of CAN on the perioperative hemodynamic response, some of them showing a relation of CAN with perioperative hypotension, whereas others did not.[6–11] These studies mainly focused on patients with diabetes mellitus (DM) and used patients without DM as ‘healthy control group’.

CAN is a well-known complication of DM.[12, 13] Although research is mainly focused on patients with DM, patients without DM are also known to develop CAN and might have the same perioperative risks as patients with DM and CAN.[6, 14] The reported prevalence of CAN is highly variable in patients with and without DM and ranges from 0 to 100%, depending on the duration of DM, glycemic control and the population studied.[7, 8, 11, 15, 16]

The aim of this study was to use the Ewing test battery and baroreflex sensitivity (BRS) to determine the prevalence of CAN in patients with and without DM undergoing major elective surgery. In addition, we set out to examine the predictive value of this set of autonomic function tests for post-induction hypotension as this could help us to identify high-risk patients at the preoperative assessment clinic.

We hypothesized that patients with DM would have a higher prevalence of CAN than patients without DM. Furthermore, we hypothesized that patients with CAN were more likely to experience post-induction hypotension and consequently would require more hemodynamic support with vasopressors.

## Methods

The protocol of this study was approved by the local ethics committee of the Academic Medical Center in Amsterdam (MEC 2014_242). The study was conducted conform guidelines of good clinical practice and the Declaration of Helsinki.[17] Written informed consent was obtained from all patients before participating in this study. Our trial was registered on 24 November 2014 in the Dutch trial registry (www.trialregister.nl #4976). This manuscript adheres to the STROBE guidelines.

We conducted a single center prospective cohort study. Participants were included between November 2014 and March 2017. Patient characteristics of adult patients scheduled for elective cardiac or major abdominal surgery were collected at the preoperative assessment clinic. Exclusion criteria were: cardiac rhythm other than sinus rhythm, Parkinson’s disease, pure autonomic failure (formerly called idiopathic orthostatic hypotension), multiple system atrophy with autonomic failure (formerly called Shy-Drager syndrome), Addison’s disease and hypopituitarism, pheochromocytoma, peripheral autonomic neuropathy (e.g., amyloid neuropathy, idiopathic autonomic neuropathy), known cardiomyopathy, extreme left ventricle hypertrophy [18], left ventricular ejection fraction < 30% [18] and proven or suspected allergy for any of the medication used during induction of anesthesia.

### Protocol

One day before surgery, continuous hemodynamic variables were assessed with the ^cc^Nexfin monitor (Edwards Lifesciences Corporation, Irvine, CA, USA) during autonomic and peripheral nervous system testing. The ^cc^Nexfin has been validated [19, 20] and used before to assess cardiovascular autonomic function.[21, 22] Ewing’s battery of tests originally consisted of five autonomic function tests,[23] however we omitted the sustained handgrip test in our series of tests, as this test was found to have limited diagnostic power.[24]

Autonomic function tests:[23]

1. *Paced breathing*: the patient was asked to take deep breaths with a frequency of 6 per minute for 1 minute. Heart rate (HR) variability between inspiration and expiration was measured. A difference in HR ≥ 15 beats per minute between inspiration and expiration was considered normal, a difference in HR ≤ 10 beats per minute was an indication of parasympathetic neuropathy and considered abnormal. A difference in HR between 11 and 14 beats per minute was considered borderline.
2. *Valsalva Maneuver*: the patient was asked to blow through a mouthpiece with a small leakage of 16 gauge and to maintain a pressure of 40 mmHg for 15 seconds. The leakage ensures an open glottis during the procedure. In normal subjects, tachycardia arises during these 15 seconds of strain with a subsequent vasoconstriction. After release of strain a hypertensive response and reflex bradycardia is observed. In patients with both sympathetic and parasympathetic neuropathy, little change is seen in HR and blood pressure. We calculated the ratio between the longest interbeat interval after release and the shortest interbeat interval during strain. A ratio of 1.21 or more was considered normal, a ratio of 1.20 or less was considered abnormal.
3. *30*:*15 ratio*: patients were asked to stand up from a supine position. In healthy subjects, tachycardia after 15 heart beats with a subsequent bradycardia after 30 heart beats is expected. An abnormal response shows a decrease in 30:15 ratio, which is due to parasympathetic neuropathy. A ratio of 1.04 or more was considered normal, a ratio of 1.01 to 1.03 was considered borderline and a ratio of 1.00 or less was considered abnormal. Because these time constants are not equal for every subject, the shortest interbeat interval around the 15^th^ beat and the longest interbeat interval around the 30^th^ beat were used for this calculation.[25]
4. *Orthostatic response*: the patients were asked to stand up from a supine position and to remain standing for three minutes. If blood pressure remains low after three minutes of standing, this is an indication for sympathetic neuropathy. A fall in systolic blood pressure (SBP) of 10 mmHg or less after three minutes was considered normal. A fall in SBP of 11–29 mmHg after three minutes was considered borderline and a fall in SBP of 30 mmHg or more after three minutes was considered abnormal.

Baroreflex sensitivity (BRS)

1. To calculate BRS, thirty seconds of data during paced breathing at 6 min^-1^ were extracted and equidistantly resampled at 0.1 Hz. SBP (in mmHg) was plotted against the interbeat interval in milliseconds (inverse of HR). There is a small latency between variations in systolic blood pressure and HR. We shifted the HR tracing 1–3 seconds to obtain the highest correlation (Pearson R) between SBP and HR. The slope of the linear regression line between the interbeat interval and SBP equals BRS expressed as ms mmHg^-1^. Higher values indicate better autonomic function. [26, 27]

Anesthesia was induced according to a standardized regimen with propofol (range 0.8 to 2.5 mg kg^-1^), sufentanil (range 0.2 to 0.5 mcg kg^-1^) and rocuronium (range 0.5 to 1.0 mg kg^-1^). Furthermore, when an epidural catheter was placed, only the local anesthetic test dose (40 mg lidocaine) was administered over the epidural catheter, immediately after its placement. During the first 10 minutes after induction of general anesthesia, no medication was administered via the epidural catheter.

Intra operative hemodynamic variables from T0 (start induction) to T10 (10 minutes after induction) and from T30 to T60 were collected from the electronic perioperative charts, containing hemodynamic measurements at least every 5 minutes, but in the majority of cases every minute. The surgical incision was part of this 30 minute window. Additionally, the use of vasopressor or inotropic support during surgery and in the postoperative period was collected via electronic chart review.

### Outcome measures

As primary outcome measure we calculated the prevalence of CAN in patients with or without DM and used a modified version of the classification of CAN originally proposed by Ewing. [23] In the version proposed by Ewing, early and definite stage are only diagnosed if the HR tests are abnormal and the orthostatic test normal. If the orthostatic test was borderline or abnormal it was diagnosed as *abnormal stage*. A relatively large group of patients were diagnosed with *abnormal stage*. Therefore we decided not to distinguish between the HR and orthostatic tests: *normal stage*: all tests normal, or one test borderline. *Early stage*: abnormal response to one of the tests with or without one borderline test or two tests borderline (either HR or orthostatic). *Definite stage*: abnormal response to two tests with or without one borderline test (HR or orthostatic). *Severe stage*: abnormal response to two of the three HR tests plus abnormal response to the orthostatic test. Additionally, the BRS was calculated to express HR variability in response to blood pressure on a continuous scale.

Our other main outcome measure was the difference in the incidence of *severe post-induction hypotension*, comparing patients with and without CAN. *Severe post-induction hypotension* was defined as a mean arterial pressure (MAP) of < 50 mmHg or a ≥ 50% decrease in MAP from baseline. This is based on the recent publication by Salmasi et al,[5] showing that these values were associated with a higher odds for developing acute kidney injury (AKI) or myocardial injury after non-cardiac surgery (MINS) when they occurred for > 1 minute.[5] This is a slight modification of our original definition (MAP<55 mmHg,www.trialregister.nl #4976), because the paper of Salmasi was published after trial registration. Baseline blood pressure was defined as the blood pressure measured at the preoperative assessment clinic. In case of a missing value, we used the blood pressure measured by a nurse on the ward one day prior to surgery.[28] We selected T0 to T10 (10 minutes), as timeframe for the induction of anesthesia.

As secondary outcome measures during induction of anesthesia we evaluated *mild post-induction hypotension*: MAP < 65 mmHg. A MAP below 65 mmHg for more than 13 minutes was also associated with greater odds in developing AKI or MINS.[5] Change in SBP, diastolic blood pressure (DBP), MAP and HR when the trachea was intubated and the need for phenylephrine and ephedrine during induction of anesthesia were assessed.

In addition we evaluated the average SBP, DBP, MAP and HR during the ‘stable phase of anesthesia’ (maintenance of anesthesia). Furthermore, the mean absolute blood pressure change was calculated as a measure of intraoperative blood pressure swings. Intraoperative hypotension was defined as a MAP < 65 for more than 13 minutes.[5] Low systolic blood pressure (LSBP) was defined as a systolic blood pressure <90 mmHg at any time during the stable phase of anesthesia. To avoid bias from intraoperative events (for example cardiopulmonary bypass or significant surgical blood loss) we chose T30 to T60 as timeframe for the ‘stable phase of anesthesia’, to assess intraoperative hemodynamic variables. If initiation of cardiopulmonary bypass was within these 30 minutes, data collection was ceased at that point. The total intraoperative dose of norepinephrine (mcg kg^-1^ min^-1^), total postoperative dose of norepinephrine (mcg kg^-1^ min^-1^) and duration of cardiovascular support (in hours) was determined.

### Sample size

A previous study by Knuttgen et al. showed that 72.2% of patients with DM and evidence of CAN experienced hypotension after induction, compared to 25% of patients without DM.[10] Assuming a power of 80% and a significance level of 0.05 we needed a minimum sample size of 14 patients per group to detect such a difference. Assuming that we would diagnose a form of CAN in 50% of patients with DM[9] with a drop-out of 5%, our minimal sample size was 45 patients (30 with DM and 15 without DM).

In order to be able to also perform a subgroup analysis between cardiac and abdominal surgery, we aimed for the inclusion of 90 patients: 45 patients (30 with DM, 15 without DM) undergoing cardiac surgery and 45 patients (30 with DM, 15 without DM) undergoing abdominal surgery.

### Data handling and statistical analyses

Data were extracted, encrypted and stored for offline analysis. Data were manually checked for quality and tests with artefacts (non-sinus rhythm during the autonomic function tests, evidence of poor calibration of the ^cc^Nexfin during the measurements) were rejected. Hereafter, data were analyzed in Matlab (2007b, MathWorks, Natick, MA, USA). If data from one test was not analyzable, we considered this test to be normal, knowing that this would lead to a possible underestimation of the prevalence of CAN. In case the anesthesiologist did not comply with the standardized induction protocol, patients were excluded from the analyses.

Patient characteristics were listed for all patients and separately for patients with and without DM. The presence of diabetes was assessed via chart review and verified with the patient. Differences between the two groups were assessed with a Student’s t-test or Mann Whitney-U test, depending on the distribution of the data. Normality was evaluated with the Shapiro-Wilk test. The BRS calculated from the paced breathing was plotted against the stages of CAN, to evaluate their level of agreement. Univariate analysis was done with a Chi-square test or non-parametric test, to evaluate the association between CAN and the hemodynamic variables. The mean absolute blood pressure change was defined as the absolute change between two adjacent blood pressure measurements divided over the time in which they were measured. In other words: it is the sum of all absolute blood pressure differences divided by 30 minutes (mmHg min^-1^).[29] In 16 of 82 patients, blood pressure was measured every three minutes instead of every minute, and missing data was imputed with a linear coefficient. To identify factors affecting the probability of having mild or moderate CAN we performed a multinominal regression analysis, correcting for age, gender, use of beta-blockers, use of ACE-inhibitors or AT2-antagonists and diabetes. We performed a logistic regression analysis to identify potential confounders for post-induction hypotension and mild hypotension. We corrected for age, gender, use of beta-blockers, use of ACE-inhibitors or AT2-antagonists, presence of CAN, propofol induction dose and perioperative fluid balance. Receiver operating characteristic (ROC) curves were plotted to evaluate the association between BRS and severe or mild post-induction hypotension. Reported p-values are 2-sided. A p-value < 0.05 was assumed to indicate statistical significant differences.

## Results

**Fig 1:**
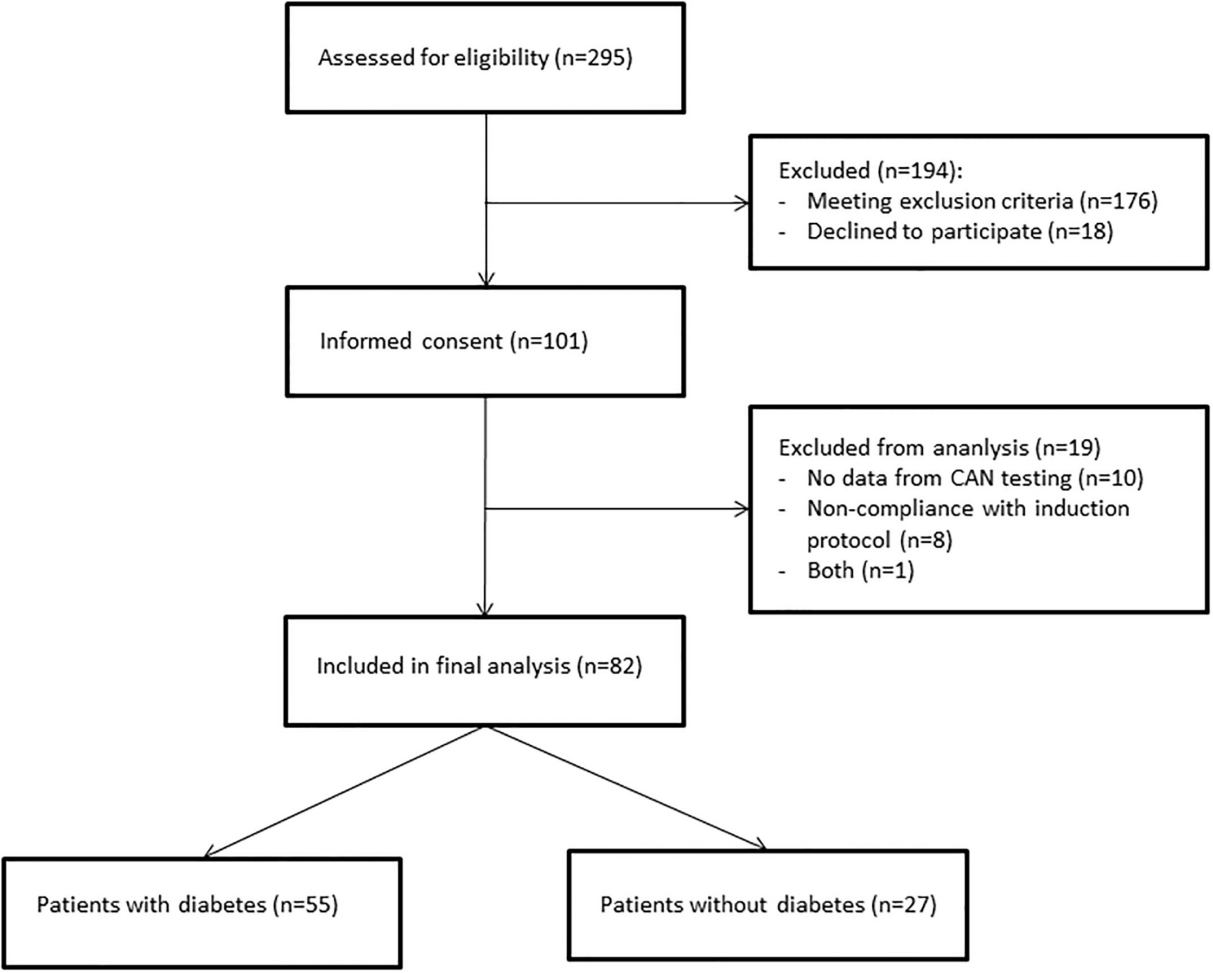
Flow chart of study.

We assessed 295 patients for potential inclusion in this study. In total, 101 patients consented and underwent autonomic function testing. Nineteen patients were subsequently excluded due artefacts in the autonomic function tests or non-compliance with the induction protocol (Fig 1). Eighty-two patients were included in the final analyses.

Patient characteristics are displayed in Table 1. Patients with DM were more often diagnosed with arterial hypertension and consequently used more beta-blockers, angiotensin converting enzyme inhibitors and angiotensin 2 receptor blockers.

**Table 1:** Patient characteristics.

	All patients (n = 82)	Diabetes + (n = 55)	Diabetes − (n = 27)	p-value
**Male**	58 (70.7)	36 (65.5)	22 (81.5)	.134
**Age (years)**	63 (11)	64 (10)	62 (12)	.499
**ASA I**	2 (2.4)	-	2 (7.4)	.064
**ASA II**	34 (41.5	21 (38.2)	13 (48.1)	
**ASA III**	46 (56.1)	34 (61.8)	12 (44.4)	
**Hypertension**	43 (52.4)	35 (63.6)	8 (29.6)	.**004**
**Myocardial infarction**	20 (24.4)	16 (29.1)	4 (14.8)	.157
**Aorta valve stenosis**	18 (22.0)	10 (18.2)	8 (29.6)	.239
**Malignancy**	27 (32.9)	17 (30.9)	10 (37.0)	.579
**ACE/AT2 inhibitors**	35 (42.7)	28 (50.9)	7 (25.9)	**.032**
**Beta blockers**	35 (42.7)	29 (52.7)	6 (22.2)	**.009**
**Calcium channel blockers**	15 (18.3)	13 (23.6)	2 (7.4)	.126
**Diuretics**	16 (19.5)	14 (25.5)	2 (7.4)	.075
***Baseline haemodynamic variables***				
**Systolic blood pressure (mmHg)**	141 (20.6)	141 (19.7)	142 (22.8)	.821
**Diastolic blood pressure (mmHg)**	80 (10.2)	80 (10.5)	82 (9.7)	.392
**Mean arterial pressure (mmHg)**	101 (12.0)	100 (12.2)	102 (11.9)	.540
**Heart rate (beats min**^**-1**^**)**	74 (11.9)	76 (11.9)	72 (11.5)	.181
**Diabetes**	55 (67.1)	-	-	-
**Duration of diabetes (median [IQR],years)**	7.5 (4–17)	7.5 (4–17)	-	-
**HbA1C (median [IQR],mmol mol**^**-1**^**)**	53 (45–67)	53 (45–67)	-	-
**Treated with insulin**	28 (34.1)	28 (50.9)	-	-
**Peripheral neuropathy**	23 (28.0)	22 (40.0)	1 (3.7)	**.003**
**Retinopathy**	2 (2.4)	2 (3.6)	-	**-**
**Nephropathy**	6 (7.3)	6 (10.9)	-	**-**
**Fasting glucose (median [IQR], mmol l**^**-1**^**)**	-	7.7 (6.7–9.5)	5.8 (5.4–8.2)	**<.001**
***Type of surgery***				.941
**Cardiac surgery**	39 (47.6)	26 (47.3)	13 (48.1)	
**Abdominal surgery**	43 (52.4)	29 (52.7)	14 (51.9)	
**Duration of surgery (min)**	222 (78)	215 (73)	236 (87)	.258

Values are: number (%) or mean (SD) unless otherwise specified. (ACE/AT2) angiotensin converting enzyme / angiotensin 2 receptor. (HbA1c) glycosylated haemoglobin, (IQR) interquartile range.

**Fig 2:**
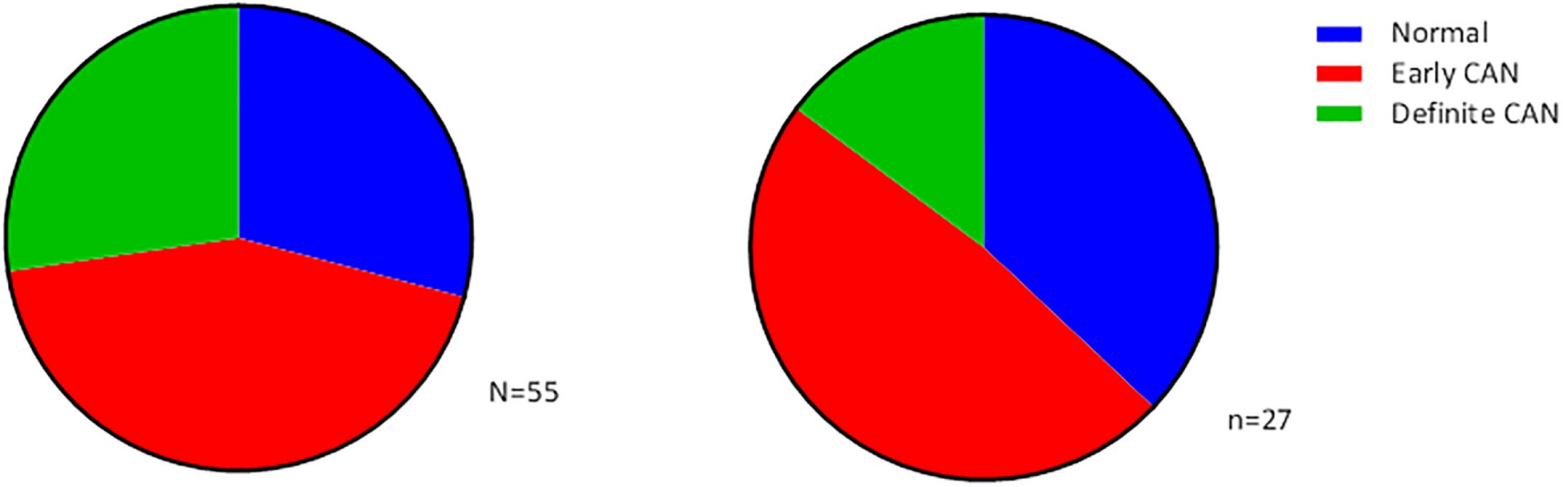
Prevalence of CAN in patients with and without DM. Left panel: patients with DM (n = 55), right panel: patients without DM (n = 27) p = 0.437.

**Fig 3:**
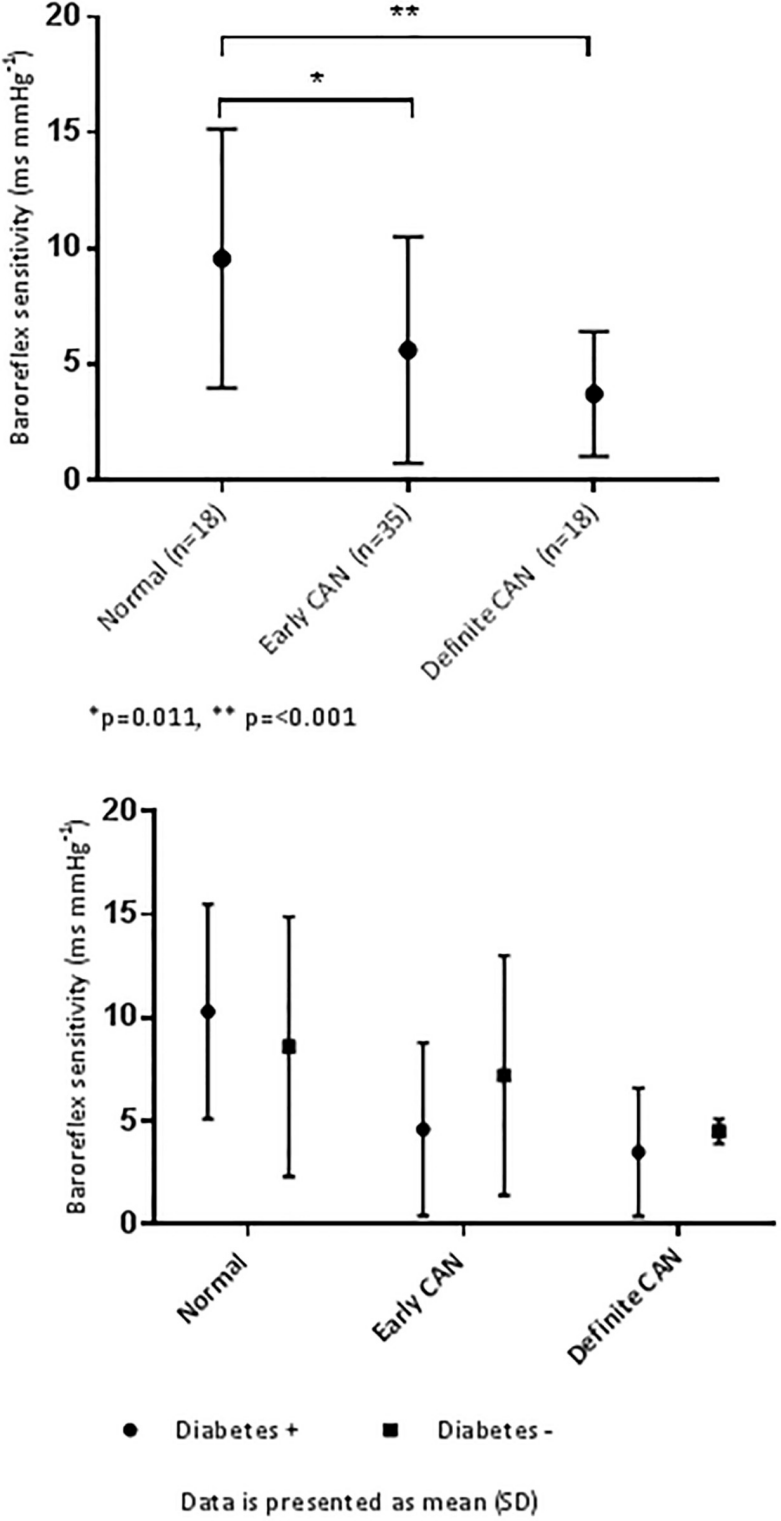
Level of agreement between stages of CAN and the baroreflex sensitivity. Upper panel: including all patients, Lower panel: separate for patients with and without DM.

There was no difference in the prevalence of CAN between patients with or without DM (p = 0.437). Out of the patients with DM, 39 (71%) had either early or definite CAN. Out of the patients without DM, 17 (63%) had either early or definite CAN (Fig 2). We did not identify patients with severe stage of CAN. The BRS was in agreement with the different stages of CAN (Fig 3), there was no difference in BRS for patients with or without DM. Multinominal regression analysis identified higher age and male gender, but not diabetes nor beta-blocker therapy, as possible predictors for having mild or moderate CAN (data not shown).

Univariate analyses showed that patients with CAN did not show an increased incidence of severe or mild post-induction hypotension compared to patients without CAN (21% vs. 19%, p = 0.819 and 46% vs. 31%, p = 0.180 respectively), nor were there any other differences in hemodynamic post-induction variables between patients with and without CAN (Table 2). Multivariate logistic regression analysis showed that the use of beta-blockers was associated with severe and mild hypotension. (OR 4.7, 95%CI 1.0–21.6, p = 0.048 and OR 12.1, 95%CI 2.7–55.1, p = 0.001 respectively). Using ACE-inhibitors or AT2-antagonists was found to be protective for mild hypotension (OR 0.2 95%CI 0.04–0.8, p = 0.027). The ROC curve showed that the BRS was not associated with severe nor mild post-induction hypotension (AUC 0.53, 95%CI 0.34–0.72 and AUC 0.42, 95%CI 0.28–0.56). In other words, the BRS had a low sensitivity and specificity and was thus not accurate enough to predict post-induction hypotension.

**Table 2:** Univariate analysis association CAN and post-induction haemodynamic variables.

	Normal (n = 26)	Mild CAN (n = 39)	Definite CAN (n = 17)	p-value
Diabetes	16 (61.5)	24 (64.9)	15 (78.9)	.467
Severe post-induction hypotension	5 (19.2)	8 (21.6)	4 (21.1)	.973
Mild post-induction hypotension	8 (30.8)	16 (43.2)	10 (52.6)	.325
Propofol induction dose (mg)	129 (56)	133 (52)	120 (44)	.467
Delta SBP post-intubation (mmHg)	20 (5–44)	20 (7–55)	9 (1–50)	.531
Delta MAP post-intubation (mmHg)	18 (3–31)	9 (1–34)	8 (3–34)	.926
Delta DBP post-intubation (mmHg)	15 (2–30)	10 (1–28)	10 (2–27)	.975
Delta HR post-intubation (beats min^-1^)	14 (5–19)	8 (3–20)	13 (0–20)	.751
Patients receiving phenylephrine or ephedrine post-induction	9 (34.6)	9 (24.3)	8 (42.1)	.371
Dose phenylephrine post-induction (microgram)	125 (50)	179 (99)	133 (52)	.443
Dose ephedrine post-induction (milligram)	8.0(2.74)	6.25 (1.76)	8.3 (2.89)	.679
Fluid balance (ml)	1157 (883)	1268 (1512)	1452 (961)	.765

Values are number(%), median (25^th^–75^th^ percentile) or mean (SD). (SBP)systolic blood pressure, (MAP) mean arterial pressure, (DBP), diastolic blood pressure, (HR) heart rate

Intraoperative hypotension occurred in 9 (16%) patients with CAN and 4 (15%) patients without CAN (p = 0.937). Also, the incidence of low systolic blood pressure did not differ between patients with or without CAN (61% vs. 62%). Furthermore, the intraoperative hemodynamic variables did not differ between patients with or without CAN (Table 3). Patients with moderate CAN received more norepinephrine perioperative compared to patients with mild CAN or normal autonomic tests (0.07 mcg kg^-1^ min^-1^ (0.05–0.08) vs. 0.03 (0.01–0.07) vs. 0.02 (0.01–0.06) respectively, p = 0.001).

**Table 3:** Univariate analyses, association of CAN and intraoperative haemodynamic variables for cardiac and abdominal surgery.

	**Normal (n = 13)**	**Mild CAN (n = 16)**	**Moderate CAN (n = 10)**	**p-value**
**Cardiac surgery**				
Average SBP intraoperative (mmHg)	113 (12)	111 (11)	109 (13)	.705
Average MAP intraoperative (mmHg)	81 (10)	78 (6)	72 (7)	.034
Average DBP intraoperative (mmHg)	65 (10)	61 (4)	54 (6)	.005
Average HR intraoperative (beats min^-1^)	63 (11)	61 (7)	56 (10)	.159
Mean absolute SBP change (mmHg)	5.8 (2.7)	5.5 (1.7)	6.7 (2.7)	.466
Mean absolute DBP change (mmHg)	3.8 (2.2)	3.1 (0.9)	3.2 (1.4)	.784
Mean absolute MAP change (mmHg)	4.2 (2.1)	3.9 (1.0)	4.4 (1.8)	.468
Mean absolute HR change (beats min^-1^)	2.4 (1.5)	2.4 (1.5)	2.5 (2.6)	.993
Patients receiving norepinephrine perioperative	12 (92.3)	16 (100)	10 (100)	.243
Norepinephrine perioperative (median [IQR] mcg kg^-1^ min^-1^)*	0.02 (0.01–0.06)	0.03 (0.01–0.07)	0.06 (0.02–0.11)	.073
Patients receiving norepinephrine postoperative	8 (66.7)	7 (63.6)	5 (62.5)	.979
Norepinephrine postoperative (median [IQR] mcg kg^-1^ min^-1^)*	0.04 (0.03–0.06)	0.06 (0.03–0.17)	0.07 (0.05–0.10)	.293
Duration of norepinephrine postoperative (hrs)	6.3 (5.7)	4.9 (6.5)	8.0 (9.4)	.680
**Abdominal surgery**	**Normal (n = 13)**	**Mild CAN (n = 21)**	**Moderate CAN (n = 9)**	**p-value**
Average SBP intraoperative (mmHg)	106 (12)	113 (18)	111 (14)	.421
Average MAP intraoperative (mmHg)	77 (8)	79 (11)	75 (10)	.607
Average DBP intraoperative (mmHg)	64 (9)	64 (10)	58 (9)	.241
Average HR intraoperative (beats min^-1^)	68 (10)	60 (9)	67 (9)	.065
Mean absolute SBP change (mmHg)	4.5 (1.9)	4.5 (1.9)	6.3 (1.9)	.050
Mean absolute DBP change (mmHg)	3.5 (1.6)	2.6 (1.3)	3.5 (0.9)	.469
Mean absolute MAP change (mmHg)	4.6 (1.1)	4.1 (1.2)	4.2 (0.9)	.094
Mean absolute HR change (beats min^-1^)	2.4 (1.2)	2.2 (1.3)	2.1 (1.1)	.784
Patients receiving norepinephrine perioperative	9 (69.2)	16 (80.0)	9 (100)	.286
Norepinephrine perioperative (median [IQR] mcg kg^-1^ min^-1^)*	0.02 (0.00–0.05)	0.03 (0.02–0.04)	0.07 (0.05–0.08)	.001
Patients receiving norepinephrine postoperative	1 (8.3)	n/a	2 (22.2)	.076
Norepinephrine postoperative (median [IQR] mcg kg^-1^ min^-1^)*	0.03 (0.01–0.04)	n/a	0.09 (0.07–0.35)	.083
Duration of norepinephrine postoperative (hrs)	n/a	n/a	11.3 (9.0)	.301

Values are mean (SD) or number(%) unless otherwise specified. (SBP)systolic blood pressure, (MAP) mean arterial pressure, (DBP), diastolic blood pressure, (HR) heart rate, (hrs) hours.

*The dose of norepinephrine is given as mcg kg^-1^ min^-1^ in order to correct for weight and duration of infusion.

## Discussion

Contrary to our expectation, the prevalence of CAN in surgical patients with DM was comparable to patients without DM. No difference was found in the prevalence of hypotension. However, patients with moderate CAN undergoing major abdominal surgery were more likely to require perioperative vasopressor therapy.

Apart from diabetes, also advancing age, hypertension, heart failure and previous myocardial infarction are known risk factors for developing CAN.[14, 30, 31] A significant part of our study population had at least one of these risk factors. This might explain why 60% of our patients without DM also complied with the criteria for early CAN. This emphasises that research on the relation between CAN and hemodynamic variables should not be limited to patients with DM.

As opposed to most studies from the early 90’s on CAN and perioperative hypotension, [6, 9–11] we could not establish a relation between CAN and post-induction or intraoperative hypotension. This might be due to the difference in populations studied, differences in induction protocol or differences in intraoperative blood pressure targets: The studies which found a relation between hypotension and CAN were performed in patients undergoing minor (ophthalmologic or ambulatory) surgery.[6, 9–11] Indeed, our results are in agreement with one study who also studied patients subjected to major (cardiac) surgery.[8] Especially patients undergoing cardiac surgery have numerous reasons other than CAN to become hypotensive during surgery; they often use more than one antihypertensive agent and might have a reduced myocardial function. Second, as the optimal induction protocol for patients with CAN is not known, every study had their own unique induction protocol. Finally, the field of anesthetic practice has evolved between the early 90’s and 2017. Anesthetic research and care today is more focused on preventing postoperative morbidity and mortality, and intraoperative hypotension has been related to both.[5, 32–34] There are many definitions for baseline blood pressure or intraoperative hypotension in the literature.[5, 28, 35] We assessed for three different definitions if post-induction or intraoperative hypotension was associated with CAN, as these definitions were related to postoperative morbidity.[5] Although the incidences of post-induction and intraoperative hypotension varied per definition used (15% to 62%), we could not detect any difference between patients with and without CAN for any of these definitions. Regardless of the highly variable definition of intraoperative hypotension, the anesthesiologist today might be more focused on preventing hypotension compared to 30 years ago.[5, 35, 36] In our dataset, patients with moderate CAN undergoing major abdominal surgery received significantly more norepinephrine compared to patients without or mild CAN during the perioperative period. This could have obscured a more pronounced hypotensive response to anesthesia in these patients.

Our data suggest that the BRS might be a valid replacement for Ewing’s battery of tests to assess CAN. This has been suggested before in patients with DM,[37, 38] but has never been shown for patients without DM. Previously, a cut-off value of < 6 ms mmHg^-1^ was classified as moderate depressed BRS and was found to correlate well with postoperative infections and cardiovascular morbidity in a post-hoc analysis of a randomized controlled trial.[27, 39] A preoperative BRS < 6 ms mmHg^-1^ was significantly associated with a postoperative BRS < 6 ms mmHg^-1^. [27] These results suggest that baseline BRS might be a good marker for postoperative morbidity. It would therefore be of interest to prospectively measure the BRS preoperatively, intraoperatively and postoperatively combined with the assessment of postoperative complications. Such a study might answer the question whether the BRS can identify patients in need for a more extensive postoperative monitoring. Unfortunately, our study lacks power to be able to comment on postoperative outcomes.

A limitation of our study is the lack of standardization of a target MAP and when to use vasopressors or inotropic agents, which could have biased our results. However, the induction of anesthesia was standardized. Furthermore, we had to exclude 11 patients based on insufficient quality of the beat-to-beat data during the autonomic function tests, as well as eight patients based on non-compliance with the induction protocol. Nonetheless, the patient characteristics of the excluded patients were comparable with the study population and we have no reason to believe that these 19 patients would have altered our results. Lastly, our sample size calculation was based on Knuttgen et al.[10] reporting a large difference in the incidence of intraoperative hypotension (75% vs 25%). We did not detect this huge difference in intraoperative hypotension (61% vs. 62%) when using the same definition as Knuttgen et al., nor when using any of the other definitions. Given the current incidence of intraoperative hypotension, we would need 248 cases and 124 controls to be able to detect such a small difference. In addition, the study was sufficiently powered, as we needed 14 patients per group (healthy, CAN with DM and CAN without DM) for our main analyses and in the end included 82 patients, with a higher incidence of CAN than expected.

To summarize, 65 to 70% of patients presenting for major surgery had mild to moderate CAN, regardless of their diagnosis of DM. Diagnosing CAN did not identify patients at risk for post-induction or intraoperative hypotension. However, moderate CAN was associated with higher vasopressor requirements in major abdominal surgery. Whether preoperative autonomic dysregulation is predictive for the risk of *post*operative hemodynamic instability or complications remains an open question.

## Supporting information

S1 File (DOC)

S2 File (PDF)
