# Supplementary material for: The prevalence of cardiovascular autonomic neuropathy and its influence on post induction hemodynamic variables in patients with and without diabetes; A prospective cohort study
Source: PLoS One. 2018 Nov 26;13(11):e0207384. doi: 10.1371/journal.pone.0207384 (PMC6261040; doi:10.1371/journal.pone.0207384)
Supplement: S2 File — (PDF) [file pone.0207384.s002.pdf]

Diabetic cardiovascular Autonomic Neuropathy in patients undergoing major abdominal and cardiothoracic Surgery

DANS study

(Version 3.0 04-12-2015)

Protocol title: Diabetic cardiovascular Autonomic Neuropathy in patients undergoing major abdominal and cardiothoracic surgery

**Protocol ID**	**DANS study**
**Short Title**	**Diabetic Autonomic Neuropathy**
**Version**	**3.0**
**Date**	**04-12-2015**
**Coördinating investigator / Project leader**	Drs. J.A.W. Polderman<br>Department of Anaesthesiology<br><br>Dr. J. Hermanides<br>Department of Anaesthesiology<br><br>Prof. Dr. B. Preckel Department of Anaesthesiology,<br><br>Academic Medical Centre, University of Amsterdam, Postbus 22660, 1100 DD Amsterdam, the Netherlands
**Principal investigator(s)**	Prof. Dr. B. Preckel,<br>Department of Anaesthesiology,<br>Academic Medical Centre,<br>University of Amsterdam,<br>Postbus 22660, 1100 DD Amsterdam,<br>the Netherlands
**Sponsor**	Prof. Dr. W.S. Schlack<br>Department of Anaesthesiology, Academic Medical Centre AMC, Postbus 22660, 1100 DD Amsterdam
**Independent Physician**	Dr. M.F. Stevens<br>Department of Anaesthesiology, Academic Medical Centre AMC, University of Amsterdam, Postbus 22660, 1100 DD Amsterdam
**Laboratory sites**	**Department of Experimental cardio physiology**
**Pharmacy**	**Not applicable**

PROTOCOL SIGNATURE SHEET

Name	Signature	Date
**For non-commercial research, Head of Department of Anaesthesiology:**<br>*prof. dr. W.S. Schlack*		
**Head of Department of cardio-thoracic surgery:**<br>*Prof. dr. B.A.J.M. de Mol*		
**Coordinating Investigator**<br>*Dr. J. Hermanides*		
**Principal Investigator:**<br>*prof. dr. B. Preckel*		

TABLE OF CONTENTS

1. INTRODUCTION AND RATIONALE 7

2. OBJECTIVES..8

3. STUDY DESIGN 8

4. STUDY POPULATION..8

5. METHODS 9

6. STATISTICAL ANALYSIS 12

7. SAFETY REPORTING...12

8. ETHICAL CONSIDERATIONS 14

9. ADMINISTRATIVE ASPECTS AND PUBLICATION...16

10 REFERENCES...18

LIST OF ABBREVIATIONS AND RELEVANT DEFENITIONS

**ABR**	**ABR form (General Assessment and Registration form) is the application form that is required for submission to the accredited Ethics Committee (ABR = Algemene Beoordeling en Registratie)**
**AE**	**Adverse Event**
**AR**	**Adverse Reaction**
**CA**	**Competent Authority**
**CCMO**	**Central Committee on Research Involving Human Subjects**
**CV**	**Curriculum Vitae**
**IC**	**Informed Consent**
**METC**	**Medical research ethics committee (MREC); in Dutch: medisch ethische toetsing commissie (METC)**
**(S)AE**	**Serious Adverse Event**
**Sponsor**	**The sponsor is the party that commissions the organisation or performance of the research, for example a pharmaceutical**
	**company, academic hospital, scientific organisation or investigator. A party that provides funding for a study but does not commission it is not regarded as the sponsor, but referred to as a subsidising party.**
**SUSAR**	**Suspected Unexpected Serious Adverse Reaction**
**Wbp**	**Personal Data Protection Act (in Dutch: Wet Bescherming Persoonsgevens)**
**WMO**	**Medical Research Involving Human Subjects Act (Wet Medisch-wetenschappelijk Onderzoek met Mensen**

SUMMARY

Rationale: Diabetes mellitus (DM) is associated with a two- to threefold increase in perioperative cardiovascular morbidity and mortality, compared to patients without DM. In addition to well-known complications of DM, diabetic autonomic neuropathy may contribute to the perioperative cardiovascular morbidity and mortality. Cardiovascular autonomic neuropathy (CAN) is one of the most serious forms of diabetic autonomic neuropathy. Patients with CAN are at increased risk of perioperative hemodynamic instability, cardiopulmonary arrest, (postoperative) silent myocardial infarction and postoperative mortality.

There are different tests to determine CAN. Ewing's battery of tests are simple, inexpensive and reliable bedside tests for CAN. If these bedside tests can help us to predict perioperative cardiovascular instability in patients with DM, it might be important to assess patients with DM for the presence of CAN during their preoperative screening, for more vigilant perioperative management.

Objective:

1) What is the prevalence of CAN in patients with DM scheduled for major abdominal / cardiothoracic surgery?
2) Does the severity of CAN relate to perioperative hemodynamic changes?

Study design: Prospective cohort study

Study population: Adult patients undergoing major abdominal and cardiothoracic surgery

Main study parameter/endpoints:

- The prevalence of CAN in this surgical population
- The relation between the stage of autonomic failure and hemodynamic changes

Study procedures: On the day before surgery, patients will be subjected to 4 non-invasive tests to assess diabetic CAN, there will be no invasive test performed for study purposes.

Nature and extent of the burden and risks associated with participation, benefit and group relatedness: There are no risks associated with participation of this study. Undiagnosed CAN might be detected. In general, after completion of this study, we will know the prevalence of CAN in patients with DM undergoing major abdominal and cardiothoracic surgery. Additionally, we will know if CAN is associated with hemodynamic instability, and we might therefore be able to determine for whom a more vigorous pre-assessment and anaesthesia plan might be necessary.

1. Introduction and rationale

Diabetes mellitus (DM) is associated with a two- to threefold increase in perioperative cardiovascular morbidity and mortality, compared to patients without DM.[1, 2] In addition to well-known complications of DM, diabetic autonomic neuropathy may contribute to the perioperative cardiovascular morbidity and mortality.[1, 3] Clinical signs of diabetic autonomic neuropathy include gastroparesis, impaired thermal regulation, impaired cerebral autoregulation, orthostatic hypotension and sudden death.[4-7] Autonomic failure of the cardiovascular system, known as cardiovascular autonomic neuropathy (CAN) is one of the most serious forms of diabetic autonomic neuropathy and is associated with the development of the latter three conditions.[8]

The prevalence of CAN in unselected patients with DM type 1 and type 2 is approximately 20%.[9] However, when accounting for increasing age, decreased glycaemic control and duration of DM, the prevalence can increase to 65%.[9] Patients with CAN are at increased risk of perioperative hemodynamic instability, cardiopulmonary arrest, (postoperative) silent myocardial infarction and postoperative mortality.[8, 10-13] Furthermore, perioperative hemodynamic instability increases the risk of developing postoperative renal and cardiac complications.[14]

CAN is caused by an imbalance in sympathetic/parasympathetic tone.[15] An early manifestation of CAN is the loss of heart rate variability, reflecting loss of parasympathetic tone, whereas the presence of orthostatic hypotension is seen in a more advanced stage of CAN and reflects loss of sympathetic tone.[16, 17] Previous prospective studies showed that patients with DM undergoing ophthalmological surgery experienced greater declines in heart rate and blood pressure during induction of anesthesia and more frequently required intraoperative vasopressors compared to patients without DM.[10-13] Moreover, patients with DM requiring intraoperative vasopressors had a more severe stage of CAN compared to patients with DM not requiring vasopressors.[10]

The gold standard in assessing CAN remains indirect measurements of end-organ responses in a specialized autonomic testing facility under standardized conditions.[3, 16] However, Ewing's battery of tests are simple, inexpensive and reliable bedside tests, which can assess CAN with a sensitivity and specificity of more than 90%.[18, 19] Ewing's battery of tests consists of 4 different test in which blood pressure and heart rates are measured during deep breathing, the Valsalva Maneuver and during change in position from lying down to standing up.[18] Non-invasive continuous arterial blood pressure, cardiac output and pulse rate monitoring is possible with the ccNexfin monitor (BMEYE Edwards Lifesciences) with reasonable accuracy.[19] The patient is connected to the ccNexfin by finger cuff and uses a volume clamp technique.[20] If these bedside tests can help us predict perioperative cardiovascular instability in patients with DM, it might be important to assess patients with DM for the presence of CAN during their preoperative screening, for more vigilant perioperative management.

In this study we aim to investigate the presence of CAN in diabetic patients scheduled for elective major abdominal and cardiothoracic surgery and its relation with perioperative hemodynamic changes.

2. OBJECTIVES

1) What is the prevalence of CAN in patients with DM scheduled for major abdominal / cardiothoracic surgery?

2) Does the stage of CAN relate to perioperative hemodynamic changes?

3. STUDY DESIGN

In this prospective cohort study, 30 patients with DM and 15 patients without DM undergoing cardiac surgery and 30 patients with DM and 15 patients without DM undergoing major gastro-intestinal surgery in the Academic Medical Centre will be included. We need 15 patients per group in order to compare the results, however patients with DM will be subdivided into groups based on the grade of the CAN. Therefore we will include 30 patients with DM per surgery type. In total 90 patients will be included.

4. STUDY POPULATION

4.1 Population (base)

Patients scheduled for major abdominal or cardiothoracic surgery, who meet the inclusion criteria will be asked for informed consent one day prior to surgery.

4.2 **Inclusion criteria**

- Willing and able to give written informed consent
- (DM type 1 or type 2)
- Scheduled for major gastro-intestinal- or cardiothoracic surgery
- Age 18-85 years
- Sinus rhythm

Exclusion criteria

- Parkinson's disease
- Pure autonomic failure (formerly called idiopathic orthostatic hypotension)
- Multiple system atrophy with autonomic failure (formerly called Shy-Drager syndrome)
- Addison's disease and hypopituitarism
- Pheochromocytoma
- Peripheral autonomic neuropathy (e.g., amyloid neuropathy, idiopathic autonomic neuropathy)
- known cardiomyopathy
- Extreme left ventricle hypertophy[21]
- Ejection fraction < 30%[21]
- Proven or suspected allergy for any of the medication used during induction of anaesthesia

4.3 Sample size calculation

This calculation is based on an earlier study[12] where 72.2% of the patients with DM experienced hypotension, compared to 25% of patients without DM. Assuming a power of 80% and a significance level of 0.05 we need a minimum sample size of 14 per group. In order to compare patients with diabetes with and without CAN we will aim for the inclusion of 45 patients (30 with DM, 15 without DM) undergoing cardiac surgery and 45 patients (30 with DM, 15 without DM) undergoing abdominal surgery.

5. METHODS

5.1 Main study parameter/endpoint

- The prevalence of CAN in this surgical population
- The relation between the stage of CAN and hemodynamic changes*

* Hemodynamic changes are defined as:

- Postinduction hypotension: mean arterial pressure <55 mmHg measured within first 10 minutes after start induction.
- Postinduction mean arterial pressure; lowest mean blood pressure measured within first 10 minutes after start induction.
- Postinduction blood pressure change: difference between highest mean arterial pressure before induction and lowest after induction before intubation.
- Postinduction heart rate change: difference between minimum heart rate after induction and maximum heart rate before induction.
- Maximum heart rate during tracheal intubation.
- Maximum mean arterial blood pressure during tracheal intubation.
- Perioperative inotropics; definition: total dose of inotropics (mcg/kg/hour) during surgery. Maximal dose during surgery (mcg/kg/hour)
- Perioperative hypotension: mean arterial pressure < 55 mmHg at any time during the procedure.
- Postoperative inotropics; definition: total dose (mcg/kg/hour) of inotropics after surgery.

5.2 Secondary study parameters/endpoints

- Ejection fraction postoperative compared to preoperatively in patients undergoing cardiac surgery, (pre- and postoperative transthoracic/transesophageal ultrasound images are made for standard care).
- Mean glucose during surgery, glucose increase during surgery, hyperglycaemia (>10 mmol/l) during surgery (measured for standard care).
- Plasma creatinine difference before and day 0, 1 and 2 after surgery (when measured for standard care).
- Length of ICU and/or in-hospital stay
- Length of inotropic dependent hours after surgery
- In-hospital mortality < 30 days after surgery
- Duration of DM

- Presence of other DM related complications
    - Peripheral neuropathy (assessed with the Semmes-Weinstein test and tuning fork)
    - Presence of micro-albuminuria (from treating physician)
    - Retinopathy (based on last visit to ophthalmologist)
- Relation between DM related complications and stage of CAN
- Difference in baroreflex sensitivity: awake and under anaesthesia
- Difference in blood flow in the middle cerebral artery between awake and anaesthetised patients, measured with transcranial Doppler.

5.3 Study procedures

At arrival at the hospital the day before surgery, oral and written information will be given to the patient. After written informed consent, the patient will be connected the the ccNexfin monitor, with a finger cuff to measure continuous blood pressure, heart rate and cardiac output. Subsequently, 6 different tests will be performed as described below.[18, 22, 23] The performance of these tests will take 30 to 60 minutes. No invasive measurements, like blood withdrawal, will be performed.

Semmes-Weinstein test
With the Semmes-Weinstein 5.07 monofilament a pressure of 10 gram is applied to both feet; the first, third, and fifth metatarsal heads and plantar surface of the distal hallux and third toe. The patient is asked to respond when pressure is applied.

Tuning fork test
The sense of vibration is tested on the distal hallux with a vibrating/non-vibrating tuning fork.

Heart rate tests
- R-R variation to deep breathing: the patient is asked to take deep breaths with a frequency of 6/min for 2 minutes. Heart rate variability is measured. A difference in heart rate > 15/min is considered normal, a difference in heart rate < 10/min is an indication for parasympathetic neuropathy.

- RR 30/15 response to standing: the patients will be asked to stand up from a supine position. Heart rate variability is measured using an ECG. A normal response will be tachycardia at 15 seconds with a subsequent bradycardia at 30 seconds. An abnormal response shows a decrease in 30/15 ratio, which is due to parasympathetic neuropathy.

Combined test
- Response to the Valsalva manoeuvre: the patient will be asked to blow through a mouthpiece with a tiny leakage of 16 gauge and to maintain a pressure of 40 mmHg for 15 seconds. The leakage ensures an open glottis during the procedure. In normal subjects: tachycardia will arise during these 15 seconds of strain with a subsequent vasoconstriction. After release of strain a hypertensive response and reflex bradycardia is seen. In patients with both sympathetic and parasympathetic neuropathy, little change is seen in heart rate and blood pressure. Heart rate variability: the ratio between the longest R-R interval after release and the shortest R-R interval during strain. Blood pressure: hypertensive response after release of strain.

Blood pressure test

- Orthostatic hypotension: blood pressure is measured when lying down. Thereafter the patients will be asked to stand. Blood pressure is again measured after 3 minutes of standing. If systolic blood pressure drops > 30mmHg or diastolic blood pressure drops > 10mmHg, the patient is considered to have orthostatic hypotension.

CAN will be defined as:

- Normal: all test normal, or one borderline.
- Early stage: abnormal response to one of the three heart rate tests or two borderline
- Definite stage: abnormal response to two of the three heart rate tests
- Severe stage: abnormal response to two of the three heart rate tests plus one of the two blood pressure tests or both borderline
- Abnormal stage: any other combination of abnormal responses during these tests.

During surgery:

As no data exist about the optimal induction of anaesthesia, we will standardise the induction according to good clinical practice in this institution. The anaesthesiologist will be consulted before inclusion. Induction of anaesthesia will be standardized with propofol 0.8 – 2.5 mg kg^{-1}, sufentanil 0.2 – 0.5 mg kg^{-1} and rocuronium 0.5 - 1 mg kg^{-1}. S-Ketamine, etomidate and thiopental cannot be used during induction. Anaesthetic details and hemodynamic parameters will be recorded in the CRF form. If a patient receives an epidural for pain management, the epidural will be used 10 minutes after induction, to avoid hypotension due to the vasodilating effects of the local anaesthetics within the first 10 minutes. No invasive tests will be performed for study purposes.

Before induction, the patient will be connected to the ccNexfin monitor. After induction the R-R variation to deep breathing is measured again; the patient will be ventilated with a frequency of 6 breaths per minute with the appropriate tidal volume to ensure an adequate minute alveolar ventilation. This will be done for 3 minutes.[24] After this procedure, the ventilator settings are adjusted to the normal settings by the attending anaesthesiologist and the ccNexfin monitor will be disconnected.

Postoperatively

By review of the patient’s medical charts, the following parameters will be assessed: the perioperative and postoperative dose of the inotropics, the pre- and postoperative ejection fraction, the creatinine on day 0, 1, 2 and 3, glucose values and length of ICU-stay. We will use the laboratory

measurements, taken for standard care. No invasive tests, like blood withdrawal, will be performed for study purposes.

In five patients with suspected CAN, we will perform the abovementioned tests, combined with middle cerebral artery blood velocity measurement by transcranial Doppler. Transcranial Doppler is a noninvasive technique; The pulsed Doppler transducer will be placed on the temporal bone to insonate the main stem of the ipsilateral middle cerebral artery and the contralateral anterior cerebral artery. This gives us insight in the cerebral autoregulation, awake and under anaesthesia, of these patients. This pilot data will be used to power a subsequent larger study on CAN and cerebral autoregulation in surgical patients. The hypothesis is that patients with CAN have an impaired cerebral autoregulation, which might deteriorate even more when anaesthetised, and requiring some kind of intervention.

5.4 Withdrawal of patients from the study

In accordance with the Declaration of Helsinki and other applicable regulations, a patient has the right to withdraw from the study at any time and for any reason without prejudice to his/her future medical care by the physician or at the institution. Patients may be removed from the study if any of the following events occur:

- Significant protocol violation or non-compliance
- Refusal of the patient to continue treatment and /or observations
- Decision by the Investigator or the Sponsor that termination is in the patient's best medical interest

Reasons for dropouts, if available, will be documented.

5.5 Replacement of individual subjects after withdrawal

Subjects who drop out will be replaced.

5.6 Follow-up of subjects withdrawn from treatment

The reasons for withdrawal will be accurately documented in the case report forms. If serious adverse events occur or other adverse events lead to withdrawal, the patient will be treated according to good medical practice and will be closely monitored until recovery.

5.7 Premature termination of the study

The study will be terminated after enclosure of the last patient.

6. STATISTICAL ANALYSIS

Patient characteristics will be calculated with the appropriate descriptive statistics. Between group differences will be analyzed with Students t-test or nonparametric testing, depending on the distribution of the data. The relation between hemodynamic parameters and the stage of CAN will be assessed with ANOVA-testing and multivariate regression analysis.

7. SAFETY REPORTING

7.1 Section 10 WMO event

In accordance to section 10, subsection 4, of the WMO, the sponsor will suspend the study if there is sufficient ground that continuation of the study will jeopardise subject health or safety. The sponsor will notify the accredited METC without undue delay of a temporary halt including the reason for such an action. The study will be suspended pending a further positive decision by the accredited METC. The investigator will take care that all subjects are kept informed.

7.2 Adverse and serious adverse events

Adverse events are defined as any undesirable experience occurring to a subject during the study, whether or not considered related to the investigational drug. All adverse events reported spontaneously by the subject or observed by the investigator or his staff will be recorded.

A serious adverse event is any untoward medical occurrence or effect that at any dose:

- results in death;
- is life threatening (at the time of the event);
- requires hospitalisation or prolongation of existing inpatients' hospitalisation;
- results in persistent or significant disability or incapacity;
- is a congenital anomaly or birth defect;
- is a new event of the trial likely to affect the safety of the subjects, such as an unexpected outcome of an adverse reaction, lack of efficacy of an IMP used for the treatment of a life threatening disease, major safety finding from a newly completed animal study, etc.

Due to the observational nature of this study, we will not report these (serious) adverse events in toetsingonline or to the METC. **7.2.1 Suspected unexpected serious adverse reactions (SUSAR)**

Adverse reactions are all untoward and unintended responses to an investigational product related to any dose administered.

Unexpected adverse reactions are adverse reactions, of which the nature, or severity, is not consistent with the applicable product information (e.g. Investigator's Brochure for an unapproved IMP or Summary of Product Characteristics (SPC) for an authorised medicinal product).

The sponsor will report expedited the following SUSARs through the web portal *ToetsingOnline* to the METC:

- SUSARs that have arisen in the clinical trial that was assessed by the MREC;

The remaining SUSARs are recorded in an overview list (line-listing) that will be submitted once every half year to the MREC. This line-listing provides an overview of all SUSARs from the study medicine, accompanied by a brief report highlighting the main points of concern. The expedited reporting of SUSARs through the web portal ToetsingOnline is sufficient as notification to the competent authority.

The expedited reporting will occur not later than 15 days after the sponsor has first knowledge of the adverse reactions. For fatal or life threatening cases the term will be maximal 7 days for a preliminary report with another 8 days for completion of the report.

7.2.2 Annual safety report

Due to the observational nature of this study, without any expected SAE's related to the study, we will not provide an annual safety report to the MREC. However we will sent an annual progress and safety report to the METC.

-

7.3 Follow-up of adverse events

All adverse events will be followed until they have abated, or until a stable situation has been reached. Depending on the event, follow up may require additional tests or medical procedures as indicated, and/or referral to the general physician or a medical specialist.

8. ETHICAL CONSIDERATIONS

8.1 Ethical guidelines

The investigator is responsible for adhering to this protocol, the Declaration of Helsinki (Fortaleza, Brazil, 2013) and the regulatory requirements.

8.2 Patient informed consent

All patients must give signed informed consent prior to enrolment in the study. In any case, Informed Consent will be obtained prior to conducting any study related test that is outside of the normal clinical practice. In the event consent cannot be obtained, the patient is not eligible for inclusion. A

patient informed consent form will be provided to each patient during their pre-assessment screening. This includes an explanation of the study, duration, expected benefits and risks or inconveniences, explanation of the alternatives, medical record access and patient anonymity, if data is used for publications or submissions for reimbursement support.

Informed consent shall be documented by the subject's dated signature as well as by the investigator's dated signature. The principal investigator may delegate this responsibility one or more of the staff members. This will be documented. The signed consent forms will be retained by the investigator and made available (for review only) to the study sponsor/auditor on request.

8.3 Ethics committee

Written approval from the Ethics Committee (EC) will be obtained prior to the start of the study.

8.4 Patient confidentiality

The investigators and institutions involved in this study will provide direct access to source data and documents only to the appropriate authorities for the purposes of monitoring, audit, Ethics Committee review or regulatory inspection. Each subject participating in the study will have agreed explicitly to such access in writing.

The investigator will ensure that no subject will be identifiable either from the final report or published results.

8.5 Protocol changes

Protocol changes will be documented as an amendment to the protocol. For changes that affect the intent or scientific soundness of the clinical study, or that may affect the welfare, safety and /or rights of the patient, the investigator will obtain approval from the applicable regulatory authorities before implementing the change. The investigator will also be required to obtain approval of the EC before implementing the changes at the site.

8.6 Benefits and risks assessment, group relatedness

Potential risks

Patients will be subjected to 6 non-invasive tests, which are performed within 30-60 minutes. No additional invasive procedures will be performed for study purposes. No risks are associated with this study.

Potential benefits

Undiagnosed CAN might be detected. In general, after completion of this study, we will know the prevalence of CAN in patients with DM undergoing major abdominal and cardiothoracic surgery. Additionally, we will know if CAN is associated with hemodynamic instability.

8.7 Compensation for injury

There are no risks associated with this study, therefore dispensation for insurance has been granted by the medical ethical committee of the AMC.

8.8 Incentives (if applicable)

Volunteers won't receive a compensation regarding this study. No other incentives will be made.

9. ADMINISTRATIVE ASPECTS AND PUBLICATION

9.1 Handling and storage of data and documents

All data entries have to be made directly in the Case Report Form (CRF). All data of each volunteer will be noted on an individual case report form. Data will be coded using a numerical code, the key to this code is only available to the research team and is stored in the trial master file.

Data will then be inserted into a database, and correctness of entries will be controlled by a second investigator. All data, including case report forms, trial master file and consent forms will be stored for 20 years after completion of the study.

9.2 Amendments

A 'substantial amendment' is defined as an amendment to the terms of the METC application, or to the protocol or any other supporting documentation, that is likely to affect to a significant degree:

- the safety or physical or mental integrity of the subjects of the trial;
- the scientific value of the trial;
- the conduct or management of the trial; or
- the quality or safety of any intervention used in the trial.

All substantial amendments will be notified to the METC and to the competent authority.

Non-substantial amendments will not be notified to the accredited METC and the competent authority, but will be recorded and filed by the sponsor.

9.3 Annual progress report

The sponsor/investigator will submit a summary of the progress of the trial to the accredited METC once a year. Information will be provided on the date of inclusion of the first subject, numbers of subjects included and numbers of subjects that have completed the trial, serious adverse events/serious adverse reactions, other problems, and amendments.

9.4 End of study report

The sponsor will notify the accredited METC and the competent authority of the end of the study within a period of 90 days. The end of the study is defined as the last patient's last visit.
In case the study is ended prematurely, the sponsor will notify the accredited METC and the competent authority within 15 days, including the reasons for the premature termination.
Within one year after the end of the study, the investigator/sponsor will submit a final study report with the results of the study, including any publications/abstracts of the study, to the accredited METC and the Competent Authority.

9.5 Public disclosure and publication policy

There are no publication disclosures. The data will be presented on national and international congresses and will be published in a peer reviewed international journal.

10. REFERENCES

1 Oakley I, Emond L. Diabetic cardiac autonomic neuropathy and anesthetic management: review of the literature. *AANA journal* 2011; **79**: 473-9
2 Vinik AI, Erbas T, Casellini CM. Diabetic cardiac autonomic neuropathy, inflammation and cardiovascular disease. *Journal of diabetes investigation* 2013; **4**: 4-18
3 McGrane S, Atria NP, Barwise JA. Perioperative implications of the patient with autonomic dysfunction. *Current opinion in anaesthesiology* 2014; **27**: 365-70

4 Kitamura A, Hoshino T, Kon T, Ogawa R. Patients with diabetic neuropathy are at risk of a greater intraoperative reduction in core temperature. *Anesthesiology* 2000; **92**: 1311-8
5 Mankovsky BN, Piolot R, Mankovsky OL, Ziegler D. Impairment of cerebral autoregulation in diabetic patients with cardiovascular autonomic neuropathy and orthostatic hypotension. *Diabetic medicine : a journal of the British Diabetic Association* 2003; **20**: 119-26
6 Vinik AI, Freeman R, Erbas T. Diabetic autonomic neuropathy. *Seminars in neurology* 2003; **23**: 365-72
7 Ziegler D. Diagnosis and treatment of diabetic autonomic neuropathy. *Current diabetes reports* 2001; **1**: 216-27
8 Jermendy G. Clinical consequences of cardiovascular autonomic neuropathy in diabetic patients. *Acta diabetologica* 2003; **40 Suppl 2**: S370-4
9 Spallone V, Ziegler D, Freeman R, et al. Cardiovascular autonomic neuropathy in diabetes: clinical impact, assessment, diagnosis, and management. *Diabetes/metabolism research and reviews* 2011
10 Burgos LG, Ebert TJ, Asiddao C, et al. Increased intraoperative cardiovascular morbidity in diabetics with autonomic neuropathy. *Anesthesiology* 1989; **70**: 591-7
11 Knuttgen D, Buttner-Belz U, Gernot A, Doehn M. [Unstable blood pressure during anesthesia in diabetic patients with autonomic neuropathy]. *Anasthesie, Intensivtherapie, Notfallmedizin* 1990; **25**: 256-62
12 Knuttgen D, Weidemann D, Doehn M. Diabetic autonomic neuropathy: abnormal cardiovascular reactions under general anesthesia. *Klinische Wochenschrift* 1990; **68**: 1168-72
13 Linstedt U, Jaeger H, Petry A. [The neuropathy of the autonomic nervous system. An additional anesthetic risk in diabetes mellitus]. *Der Anaesthesist* 1993; **42**: 521-7
14 Charlson ME, MacKenzie CR, Gold JP, Ales KL, Topkins M, Shires GT. Preoperative characteristics predicting intraoperative hypotension and hypertension among hypertensives and diabetics undergoing noncardiac surgery. *Annals of surgery* 1990; **212**: 66-81
15 Ziegler D. Diabetic cardiovascular autonomic neuropathy: prognosis, diagnosis and treatment. *Diabetes/metabolism reviews* 1994; **10**: 339-83
16 Spallone V, Bellavere F, Scionti L, et al. Recommendations for the use of cardiovascular tests in diagnosing diabetic autonomic neuropathy. *Nutrition, metabolism, and cardiovascular diseases : NMCD* 2011; **21**: 69-78
17 Vinik AI, Ziegler D. Diabetic cardiovascular autonomic neuropathy. *Circulation* 2007; **115**: 387-97
18 Ewing DJ, Martyn CN, Young RJ, Clarke BF. The value of cardiovascular autonomic function tests: 10 years experience in diabetes. *Diabetes care* 1985; **8**: 491-8
19 Keet SW, Bulte CS, Sivanathan A, et al. Cardiovascular autonomic function testing under non-standardised and standardised conditions in cardiovascular patients with type-2 diabetes mellitus. *Anaesthesia* 2014; **69**: 476-83
20 Sipkens LM, Treskes K, Ariese-Beldman K, Veerman DP, Boer C. Application of Nexfin noninvasive beat-to-beat arterial blood pressure monitoring in autonomic function testing. *Blood pressure monitoring* 2011; **16**: 246-51
21 Lang RM, Bierig M, Devereux RB, et al. Recommendations for chamber quantification: a report from the American Society of Echocardiography's Guidelines and Standards Committee and the Chamber Quantification Writing Group, developed in conjunction with the European Association of Echocardiography, a branch of the European Society of Cardiology. *Journal of the American Society of Echocardiography : official publication of the American Society of Echocardiography* 2005; **18**: 1440-63
22 Ewing DJ, Clarke BF. Autonomic neuropathy: its diagnosis and prognosis. *Clinics in endocrinology and metabolism* 1986; **15**: 855-88
23 Perkins BA, Olaleye D, Zinman B, Bril V. Simple screening tests for peripheral neuropathy in the diabetes clinic. *Diabetes care* 2001; **24**: 250-6

24 Pennekamp CW, Immink RV, Buhre WF, Moll FL, de Borst GJ. Phenylephrine versus ephedrine on cerebral perfusion during carotid endarterectomy (PEPPER): study protocol for a randomized controlled trial. *Trials* 2013; **14**: 43
